# Validating Structural Predictions of Conjugated Macromolecules in Espaloma-Enabled Reproducible Workflows

**DOI:** 10.3390/ijms26020478

**Published:** 2025-01-08

**Authors:** Madilyn E. Paul, Chris D. Jones, Eric Jankowski

**Affiliations:** Micron School of Materials Science and Engineering, Boise State University, Boise, ID 83725, USA; madilynpaul@u.boisestate.edu (M.E.P.); chrisjones4@u.boisestate.edu (C.D.J.)

**Keywords:** polymer & macromolecule science, polymer science, organic semiconductors

## Abstract

We incorporated Espaloma forcefield parameterization into MoSDeF tools for performing molecular dynamics simulations of organic molecules with HOOMD-Blue. We compared equilibrium morphologies predicted for perylene and poly-3-hexylthiophene (P3HT) with the ESP-UA forcefield in the present work against prior work using the OPLS-UA forcefield. We found that, after resolving the chemical ambiguities in molecular topologies, ESP-UA is similar to GAFF. We observed the clustering/melting phase behavior to be similar between ESP-UA and OPLS-UA, but the base energy unit of OPLS-UA was found to better connect to experimentally measured transition temperatures. Short-range ordering measured by radial distribution functions was found to be essentially identical between the two forcefields, and the long-range ordering measured by grazing incidence X-ray scattering was qualitatively similar, with ESP-UA matching experiments better than OPLS-UA. We concluded that Espaloma offers promise in the automated screening of molecules that are from more complex chemical spaces.

## 1. Introduction

Molecular simulations offer promise for the high-throughput screening of thermodynamically stable structures from families of molecules. Such screening studies can identify chemistries and conditions where the self-assembly of desired structures are optimized, and they can provide insight into identifying chemistry–structure—property relationships that are otherwise inaccessible with experimental methods [1,2,3,4,5,6]. One challenge of screening studies, particularly of polymers, is that the parameters that define the interactions between and within model molecules may not have been defined for a given “off-the-shelf” forcefield [7]. In molecular mechanics simulations, forcefields define the potential energies and therefore forces of bond, angle, torsion/dihedral, and non-bonded models of interatomic interactions [8,9]. For example, the popular Optimized Potentials for Liquid Simulations (OPLSs) forcefield has mainly been built around simulating hydrocarbons and proteins [10,11]. The General Amber Forcefield (GAFF) is optimized for small organic molecules [12].

An example of missing parameters arises when attempting to simulate the popular organic semiconductor poly(3-hexylthiophene) (P3HT). When using OPLS, we find the forcefield is missing parameter definitions for hydrogen–carbon–carbon–sulfur (H-C-C-S) dihedrals. In such cases, the simulator typically has had two options: (1) they might reuse a similar parameterization from a forcefield based on their chemical intuition, or (2) they can perform quantum chemical calculations to identify new forcefield parameters [8] that best fit the calculated potential energies. The former option has the downside of missing important physics and giving inaccurate results, while the latter option requires additional software fluency and calculation time [7]. Both options suffer from an additional shortcoming: the forcefields that result are different from the forcefields from which they were derived, which complicates data provenance and reproducibility.

Recent efforts toward making simulations more transparent, reproducible, usable, and extensible (TRUE) [13,14] have identified programmatic workflows as a best practice. In the present context, this means that simulation scripts that fully specify forcefields contribute to more reproducible simulations, even if the forcefields are custom derivatives of prior work [15]. Quantum chemical calculations could therefore be included into the programmatic specification of custom forcefields and, in principle, be shared as reproducible workflows.

**Figure 1:**
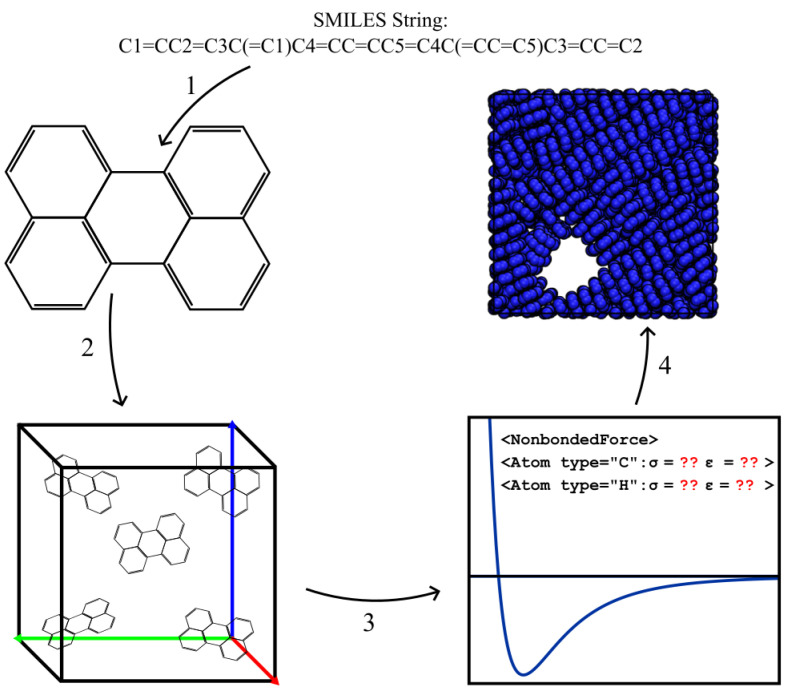
Depictions of our generalized molecular dynamics workflow. Step 1 shows the creation of an mBuild Compound from a SMILES string. In Step 2, we create our simulation object using flowerMD and PACKMOL. We employed Espalomato parameterize our molecules in Step 3 and write the forcefield file. In Step 4, we initialized the HOOMD-Blue simulation and predicted the morphology of our molecules.

However, re-running quantum chemical calculations itself introduces software dependency issues and extended runtimes that are redundant. A new tool that ameliorates this problem is Espaloma, which is an open-source package that uses graph neural networks to perceive chemical environments in a molecular graph and predict molecular mechanics forcefield parameters [7]. Espaloma, therefore, encapsulates a machine-learned forcefield designed for the investigation of biopolymers, and it has been used to accurately simulate them [16,17]. Of central importance to the present work is Espaloma’s promise to programmatically “fill in” missing parameters for organic macromolecules and polymers, enabling more TRUE screening studies without extra quantum chemical calculations (Step 3 of Figure 1). The donor–acceptor copolymers of Danielsen et al. [18] are an example set of chemistries where Espaloma could be extremely useful, enabling arbitrary sequences of monomers to be parameterized and simulated. The conjugated systems of these polymers are uncommon in the data used to train Espaloma, however, so it is not yet understood whether Espaloma parameterizations of such moieties match the expected thermodynamics and if the structure will be broadly accurate.

Here, we evaluate Espaloma for the modeling of highly conjugated macromolecules P3HT and perylene. We generated forcefield parameters using Espaloma and compared them to the “off-the-shelf” OPLS-UA and GAFF forcefields where possible. We used the Espaloma parameters to inform UA representations of the perylene and P3HT that may be directly validated against prior simulations. We compared phase diagrams and morphologies to validate and quantify Espaloma’s predictive capabilities outside its core training set [7]. A schematic of the MD workflow we implemented in this work is shown in Figure 1.

## 2. Model

**Figure 2:**
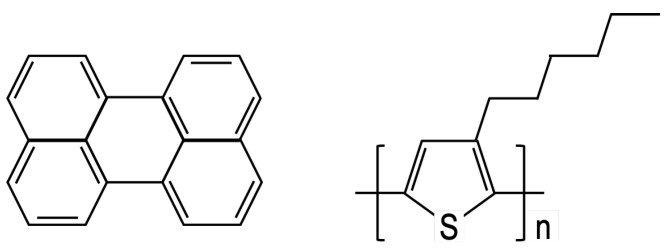
Diagram of the perylene molecule (**left**) and poly-3-hexylthiophene (P3HT) monomer (**right**).

In order to validate the present predictions against prior work, we consider the united-atom (UA) representations of perylene and P3HT, omitting long-range electrostatics as in prior work [19,20]. Spherical simulation elements were used to represent each “heavy” atom and its bonded hydrogens. Here, the heavy atoms were carbon and sulfur, which were topologically connected, as shown in Figure 2.

Chains of n=15 repeat units were used to represent P3HT oligomers. Harmonic potentials were used to model the bond stretching (Equation (1)) and angle bending (Equation (2)) between pairs and triplets of bonded atoms. $E_{bonds}$ is the potential energy of a bond, $K_{r}$ is the bond’s harmonic spring constant, *r* is the internuclear distance, and $r_{eq}$ is the equilibrium internuclear distance.

(1) $$E_{bonds}=K_{r}{\left(r-r_{eq}\right)}^{2},$$

where $E_{angle}$ is the potential energy of a bond angle, $K_{\theta }$ is the harmonic angle constant, θ is the bond angle, and $\theta_{eq}$ is the equilibrium bond angle.

(2) $$E_{angles}=K_{\theta }{\left(\theta -\theta_{eq}\right)}^{2}.$$

Cosine series were used to model proper dihedrals (Equation (3)) across the quadruplets of bonded atoms, where $E_{dihedral}$ is the potential energy of the dihedral, $K_{n}$ is the force constant of the corresponding particle, *n*, ϕ is the dihedral angle, and γ is the phase angle.

(3) $$E_{dihedral}=\sum_{n=1}^{4}\frac{K_{n}}{2}\left[1+\cos\left(n\phi -\gamma \right)\right].$$

(4) $$E_{LJ}\left(r_{i,j}\right)=4\epsilon_{i,j}\left[{\left(\frac{\sigma_{i,j}}{r_{i,j}}\right)}^{12}-{\left(\frac{\sigma_{i,j}}{r_{i,j}}\right)}^{6}\right].$$

Simulation elements that are not bonded or separated by more than three bonds within a molecule interact only via the 12-6 Lennard–Jones potential (Equation (4)) [21], which is truncated at $r_{cut}=2.5\sigma$, where σ is the length unit corresponding to the largest simulation element being represented. Here, this corresponds to sulfur (S) simulation elements for P3HT (σ=3.565 Å) and carbon (C1) elements for perylene (σ=3.380 Å). We applied the Lorentzian combination rule for sigma and a geometric combination rule for epsilon, as shown in Equation (5).

(5) $$\sigma_{i,j}=\frac{\sigma_{i}+\sigma_{j}}{2},\epsilon_{i,j}=\sqrt{\epsilon_{i}\epsilon_{j}}.$$

The parameterization of the bond, angle, dihedral, and nonbonded interaction potentials were generated via Espaloma , and we described the details and challenges with this, as shown in the Methods (Section 4).

We emphasize that the prior work modeling perylene and P3HT did not use OPLS-UA or GAFF “out-of-the-box”, and we chose the present modeling assumptions to provide the closest comparisons for validation. Forcefield parameters from OPLS-UA and TRAPPE-UA were combined for the perylene model [19], where partial charges were shown not to impact phase behavior. Likewise, the work of Ref. [20] used DFT-augmented OPLS-AA [22] to inform a UA model that omits partial charges to predict experimentally validated P3HT morphologies. Through using Espaloma to inform a UA model (termed ESP-UA here), which is compared against UA models of conjugated molecules (termed OPLS-UA) that Espaloma was not trained for, we evaluated the transferability of the Espaloma parameterizations independent of the issue of partial charges. We recognize that electrostatics are essential for many self-assembled morphologies, though exhaustive evaluation and comparison of approaches for conjugated systems is beyond the scope of the present work.

## 3. Results and Discussion

Here, we compare the ESP-UA , GAFF, and OPLS-UA parameters, which is followed by a reporting on structural differences observed between the ESP-UA-generated morphologies and the OPLS-UA morphologies generated in prior work. Details of MD protocols, characterization methods, and overall workflows are in Section 4.

### 3.1. Espaloma vs. OPLS-UA and GAFF

The nonbonded pair interaction for perylene and P3HT are presented in Table 1, where they were compared against OPLS-UA and GAFF parameters.

We first considered the LJ σ values, and we noted that OPLS-UA is somewhat of an outlier, with much larger C0 diameters, while ESP-UA and GAFF are similar to each other. The ESP-UA C1’s were slightly larger than both OPLS-UA and GAFF, while the ESP-UA C0’s were slightly smaller than GAFF. GAFF and ESP-UA were found to agree on the diameter of S atoms in P3HT. We next considered the ϵ values and noted that ESP-UA was very similar to GAFF once again, with OPLS-UA as the outlier.

Normalizing the $\epsilon_{C1}$ and $\epsilon_{C0}$ values by $\epsilon_{S}$ provides a clearer picture of the range of attractions in each model than a simple comparison of the absolute values of each ϵ. For ESP-UA, $\epsilon_{C1}/\epsilon_{S}=0.3475$, which is nearly identical to that of OPLS-UA: $\epsilon_{C1}/\epsilon_{S}=0.3437$. For C0, ESP-UA $\epsilon_{C0}/\epsilon_{S}=0.4353$, which is about 20% weaker than that of OPLS-UA: $\epsilon_{C0}/\epsilon_{S}=0.5312$. To briefly summarize, the C1 parameterizations were close-but-not identical between ESP-UA and OPLS-UA, so we expect the perylene simulations to be similar between the two models. However, because the $\epsilon_{C1}/\epsilon_{S}$ and $\epsilon_{C0}/\epsilon_{S}$ ratios and C0 σ values varied significantly between ESP-UA and OPLS-UA, it is unclear whether the P3HT morphologies and phase behavior with ESP-UA will match those previously generated with OPLS-UA.

We performed MD simulations of each molecule to compare the morphologies generated with the present ESP-UA parameterization against those previously generated with OPLS-UA. A summary of the simulation statepoints and units for each molecule is provided in Table 2. These statepoints were chosen to replicate the structures sampled in prior work from Miller et al. [20]. Each temperature range encompassed the solid–liquid transition temperatures of approximately 500 K and 550 K for P3HT and perylene, respectively. The density ranges were chosen, as well as the experimental thin-film studies for P3HT, with respect to previous MD studies [23]. We expected to observe various phases (ordered, liquid, vapor, and disordered) over this range of statepoints.

### 3.2. Perylene

**Figure 3:**
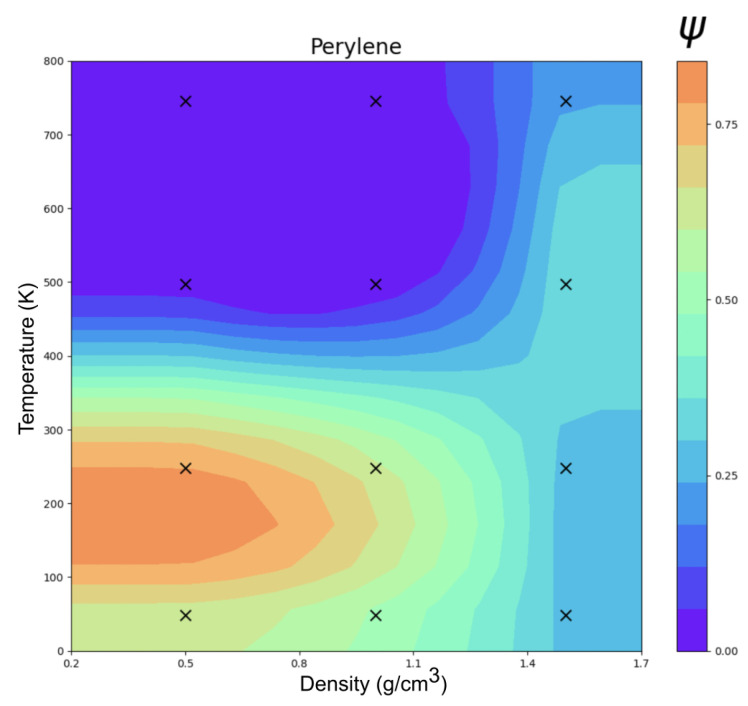
Temperature vs. density clustering in an order parameter (Ψ) phase diagram of perylene at 12 statepoints.

**Figure 4:**
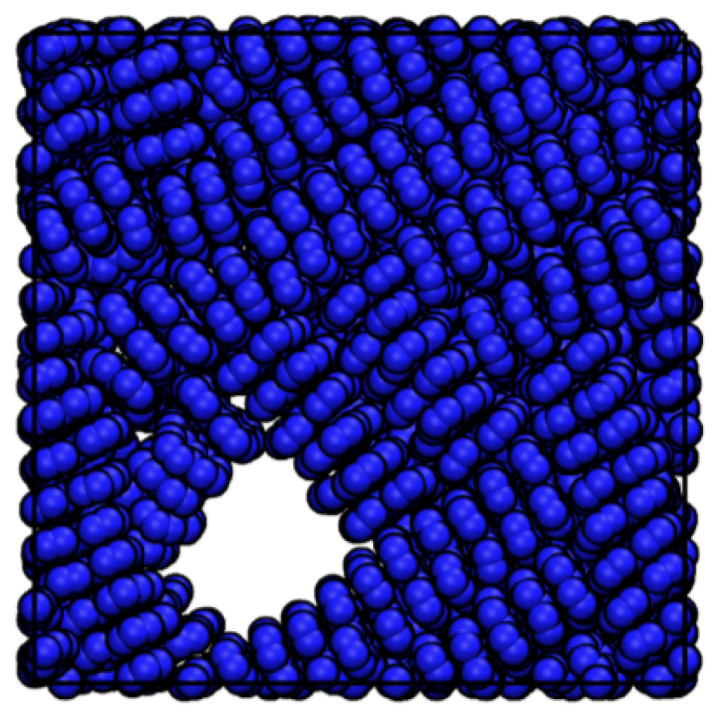
Snapshot of the perylene taken from the most ordered morphology at a density of 0.5 g/cm^3^ and temperature of 248.8 K.

The ordering (Ψ) of perylene modeled with ESP-UA as a function of temperature and density is summarized in Figure 3. Consistent with Ref. [20], the most ordered structure (T = 250 K and ρ = 0.5 g/cm^3^) exhibited significant π-stacking, which is visible in Figure 4. At lower temperatures, we observed a transition from highly ordered to less ordered as the density increased, which is also in agreement with prior work. This is due to the steric hindrance of having a high number of molecules in a restricted volume, as has also been observed in prior work.

However, the order–disorder transition temperature of 600 K in Ref. [19] and Botoshansky et al. [24] was roughly 1.26 times the 470 K observed here. This is explained by the difference in base unit between the Espaloma and OPLS-UA perylene forcefields: both models had only C1 atomtypes but $\epsilon_{OPLS-UA}=0.4602$ kJ/mol and $\epsilon_{ESP-UA}=0.3635$ kJ/mol. The ratio between these two energy units $\frac{\epsilon_{OPLS-UA}}{\epsilon_{ESP-UA}}=1.26$ matches the transition temperature discrepancy, that is, in dimensionless units, there is no significant difference in the temperature-density phase diagrams between the two forcefields. Given that the energy unit of OPLS-UA gives an order–disorder transition temperature in agreement with experiments, this suggests that the absolute ESP-UA energy units may benefit from empirical rescaling.

**Figure 5:**
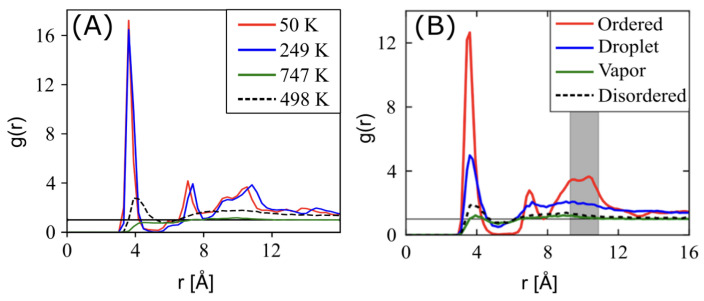
(**A**) Radial distribution function (RDF) of perylene at various temperatures at a density of 0.5 g/cm^3^, which were generated from an ESP-UA-predicted morphology. (**B**) The RDF of perylene in various phases, which was generated from an OPLS-UA-predicted morphology. The OPLS-UA RDF published by Miller et al. in Ref. [19].

Comparing the RDFs reported in Ref. [19] at a density of 1.7 g/cm^3^ against the present work shows good agreement in short-range ordering as a function of temperature. At the highest temperatures (i.e., 747 K, and shown in green in Figure 5A) we observed near-ideal gas behavior as in the vapor phase of Figure 5B, though the slightly lower first-correlation peak at 4.0 Å indicates ESP-UA has lower short-range attractions, as expected by the differences in ϵ. Disordered structures at moderate temperatures (498 K in Figure 5) have the same peak locations as in prior work, but slightly higher intensities (e.g., g(4)=3.8 vs. g(4)=1.8). As temperature was lowered further, we saw close agreement between the ordered (shown as red 50 K in Figure 5), but we also saw more ordering at 249 K (shown as blue in Figure 5) than in the droplet phases observed in prior work. Taking a closer look at the ordered (red) phases, we observed consistency across the first four local maxima locations, with slightly higher intensities in the present work and with a lower 3rd–4th peak ratio than in prior work, indicating subtle packing differences. Thus, the trend from disordered vapor phases to condensed, crystalline packings was consistent between the two forcefields, though the specific temperatures at which the transitions occurred varied due to the relative differences in ESP-UA and OPLS-UA energy scales.

**Figure 6:**
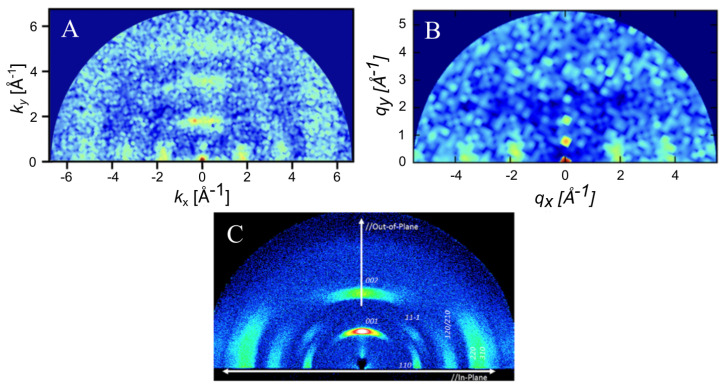
(**A**) The grazing incident X-ray scattering pattern of perylene generated from an ESP-UA morphology. (**B**) The GIXS pattern of the perylene generated from an OPLS-UA morphology. The OPLS-UA GIXS pattern published by Miller et. al. in Ref. [19]. (**C**) The experimental XRD pattern for β−perylene, which was reproduced with permission from Ishii et al. from Ref. [26]. Copyright 2014 AIP Publishing LLC. (Melville, NY, USA).

Long-range order was measured via simulated GIXS at a temperature of 250 K and a density of 0.5 g/cm^3^ (Figure 6). Bragg reflections were observed along both the x and y axes, indicating significant long-range order and close packed columns, which are observed in Figure 4. One major difference between the ESP-UA-generated GIXS pattern here and the OPLS-UA pattern in prior work is the $q_{y}$ location of the 001 reflection. From OPLS-UA, the 001 reflection occurred around $0.8\;A^{-1}$, while with the ESP-UA here, $q_{y}=1.9\;A^{-1}$, which is in better agreement with Ishii and Miyasaka [25] (Figure 6C).

To briefly summarize, modeling perylene with ESP-UA parameterization results in phase behavior and short-range ordering, which is in agreement with prior OPLS-UA work. However, long-range ordering, as measured by GIXS, matches experiments better, while the base energy unit of OPLS-UA gives better temperature correspondence.

### 3.3. Poly-3-Hexylthiophene (P3HT)

**Figure 7:**
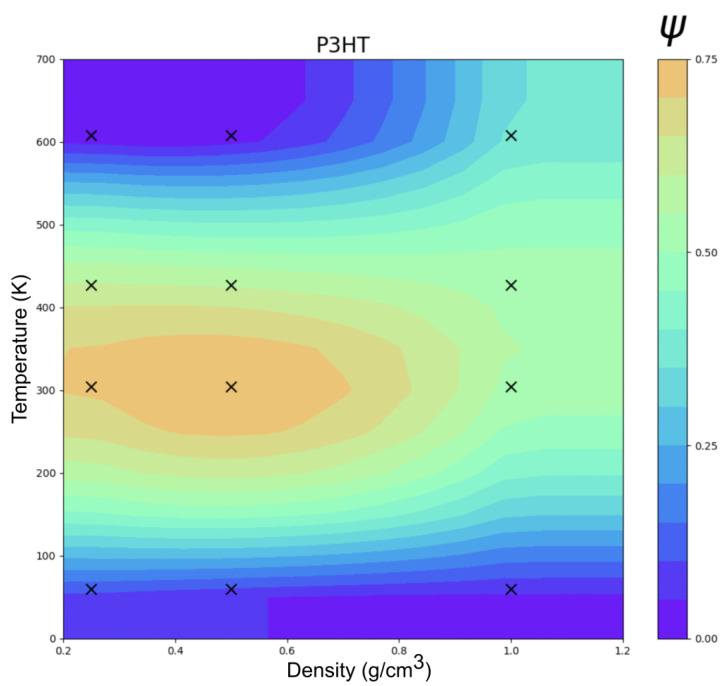
Temperature vs. density clustering in an order parameter (Ψ) phase diagram of P3HT at 12 statepoints.

Figure 7 shows the clustering order parameter (Ψ) phase diagram of P3HT as a function of temperature and density. As observed with perylene, the transition temperatures were low relative to the experiments, and this is again explained by the differences in ϵ units between ESP-UA and OPLS-UA. ESP-UA gives an ϵ of 0.4554 kJ/mol for the aliphatic side chain carbons of P3HT, while OPLS-UA uses an ϵ of 0.7113 kJ/mol. This creates more ordered side chains in the OPLS-UA simulations in comparison to the ESP-UA simulations. This observation is supported by the work conducted in reference [27], which stated that lower ϵ values for the side chains of P3HT monomers results in lamellar structures at lower temperatures than if the ϵ was higher [27]. The observed order–disorder transition temperature here of about 390 K was a factor of 1.26 lower than the experimental melting temperature of 490 K. As observed with perylene, this can be explained by the base energy unit differences between ESP-UA and OPLS-UA for sulfurs: $\frac{\epsilon_{OPLS-UA}}{\epsilon_{ESP-UA}}=1.26$. As such, we expected the equivalent temperatures to be scaled proportionally $\frac{k_{B}T_{OPLS-UA}}{\epsilon_{OPLS-UA}}=\frac{k_{B}T_{ESP-UA}}{\epsilon_{ESP-UA}}$.

**Figure 8:**
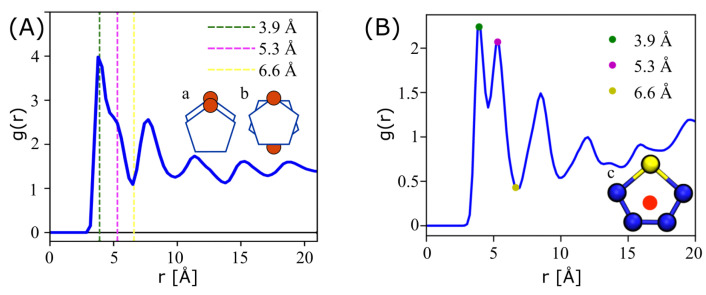
(**A**) The radial distribution function (RDF) of P3HT at a temperature of 304 K and a density of 0.5 g/cm^3^, which was generated from an ESP-UA-predicted morphology. (**B**) The RDF of P3HT generated from an OPLS-UA-predicted morphology. The OPLS-UA RDF published by Miller et al. in Ref. [20].

Figure 8 shows the radial distribution function of ESP-UA P3HT (left) and OPLS-UA P3HT (right). The ESP-UA RDF is generated at a temperature of 304 K and a density of 0.5 g/cm^3^. The three vertical dashed lines in the ESP-UA RDF correspond to the local maxima and minima highlighted in the OPLS-UA RDF. The first peak in each corresponds to the aligned π-stacking of the thiophene rings, as shown in Figure 8A, with higher g(3.9 Å) intensities in the present ESP-UA work. The second peak aligns with the anti-aligned π-stacking of the thiophene rings, as shown in Figure 8B, though the intensity relative to the first peak was found to be lower than in prior work. The OPLS-UA RDF was calculated using the geometric center of thiophene ring, as shown inset Figure 8B The ESP-UA RDF was calculated using only the S-S interactions, excluding those in the same chain. The sulfurs were chosen for the ESP-UA RDF because they are central to the ring, hold the most mass, and have the largest radius. In sum, the local ordering of P3HT in condensed phases in the present work was found to be qualitatively similar to prior work, but, as with perylene, subtle quantitative differences in the correlation peak intensities arose from the variations in the relative interaction strengths across the two models.

**Figure 9:**
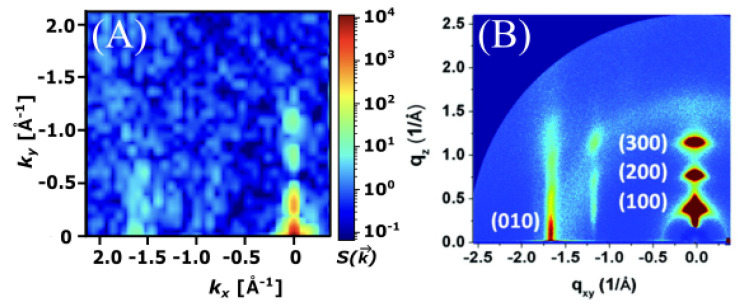
(**A**) The grazing incident X-ray scattering pattern of P3HT at 0.5 g/cm^3^ and 304 K generated using ESP-UA. (**B**) The corresponding experimental scattering pattern of P3HT. Reprinted (adapted) with permission from Ko et al., as shown in Ref. [28]. Copyright 2012 American Chemical Society.

**Figure 10:**
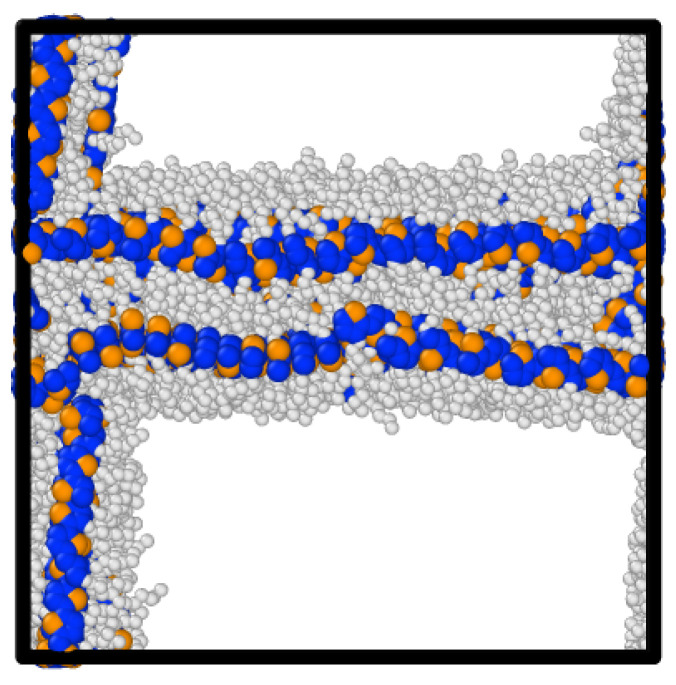
Snapshot of the P3HT’s most ordered morphology at a density of 0.5 g/cm^3^ and a temperature of 304 K.

The GIXS scattering pattern of the most ordered structure (0.5 g/cm^3^, 304 K) displayed significant correlation with the experimental scattering pattern of P3HT (Figure 9). Peaks were observed at approximately 1.65 Å^−1^ corresponding to the (010) plane, as well as with the peaks along the (100) plane, which were spaced approximately 0.3 Å^−1^ apart. This correlation between experiment and simulation confirmed that the ESP-UA was able to responsibly parameterize and represent the P3HT polymer in simulations. The lamellar structure represented by the scattering pattern in Figure 9 is shown in Figure 10.

## 4. Methods

In this section, we detail the molecular dynamics simulations performed with HOOMD-Blue [29] to predict equilibrium morphologies, describe the new tools we developed for integrating Espaloma into workflows utilizing the Molecular Simulation Design Framework (MoSDeF) [30], and detail the morphology characterization that underpins model validation.

### 4.1. Molecular Dynamics

Simulations were performed on the Fry high-performance computing cluster at Boise State University using HOOMD-Blue on NVIDIA P100 and V100 GPUs. We used signac [31] to manage the simulation workspaces and job submission. Simulation scripts are available online at https://github.com/madilynpaul/Espaloma-Validation (accessed on 2 January 2025). Equilibrium morphologies of perylene and P3HT were predicted in the canonical ensemble (constant number of particles *N*, volume *V*, and temperature *T*). N=100 for P3HT and N=250 for perylene. Periodic boundary conditions of cubic volumes were used throughout this work. We initialized our system using PACKMOL [32] within flowerMD [33] to create initial low-density volumes that were randomized at high *T* (1653 K for P3HT and 696 K for perylene) and shrinking simulation runs for $5\times 10^{6}$ time steps to the target statepoint density. Newton’s equations of motion were integrated using a two-step MTK velocity-verlet implementation of Nosé–Hoover chains [34,35], with a step size of dt=0.0003 for P3HT and dt=0.0001 for perylene. Simulation volumes were then instantaneously quenched to the statepoint temperature, as shown in Table 2, where the potential energy trajectory was analyzed to determine the onset of equilibrium. Once the potential energy had stabilized, the simulations continued until the decorrelation time measurements provided at least 50 statistically independent snapshots. We report results from single simulation runs (not averages across multiple parallel simulations), requiring between $2\times 10^{6}$ and $15\times 10^{6}$ steps for 50 independent samples.

### 4.2. Morphology Characterization

**Figure 11:**
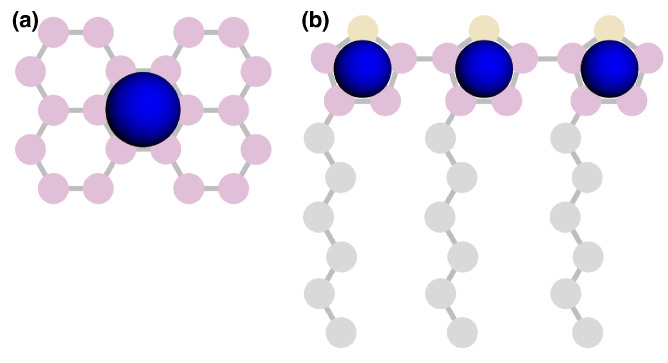
Blue spheres represent the center-of-geometry positions used for RDFs and clustering criteria. Perylene (**a**), and P3HT (**b**).

Equilibrium morphologies were quantified using radial distribution functions (RDF) and simulated grazing-incident X-ray scattering (GIXS) implemented in freud [36]. RDFs were calculated between perylene centers of mass (Figure 11a), and the sulfur of the thiophene rings was calculated for P3HT (Figure 11b). Both the RDF and GIXS analyses were averaged over the last 20 independent snapshots of each simulation.

To compare against prior work, we calculated an order parameter Ψ, as was used by Miller et al. [20], which measures the fraction of perylene molecules—or monomers for P3HT—belonging to clusters of at least size 6. Two perylene molecules—or two thiophene rings for P3HT—were considered clustered if their centers were within 6 Å and the best-fit planes through each moiety were within 10 degrees of being parallel. The code for calculating RDFs, GIXS, Ψ, and phase diagrams is available at https://github.com/madilynpaul/Espaloma-Validation.

### 4.3. Integrating Espaloma Parameterization into MoSDeF

**Figure 12:**
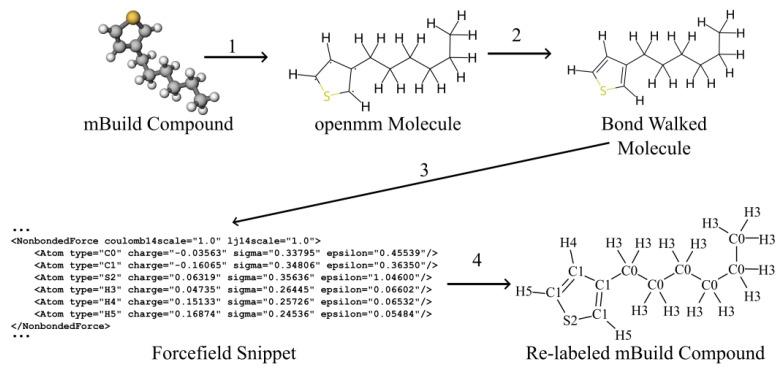
Overall Espaloma-MosDeF workflow. (1) mBuild compounds were used to create the topology of an openMM molecule, (2) BondWalker used octet rules to determine the double bonds in the openMM molecule. (3) Espaloma generated forcefield parameters for the openMM molecule. (4) The ESP-UA forcefield was used to re-type the mBuild compound for use in HOOMD-Blue simulations.

Our simulation workflows are built around MosDeF tools [30], particularly mBuild [37], whose mbuild Compound objects are incompatible with the openMM Molecule objects expected by Espaloma. To incorporate Espaloma into MoSDef workflows, we developed a helper function termed “BondWalker” that can convert mbuild Compounds into openMM Molecules (Figure 12). This workflow fits into the overall MD workflow (Section 3.2, Figure 1) within Step 4.

**Figure 13:**
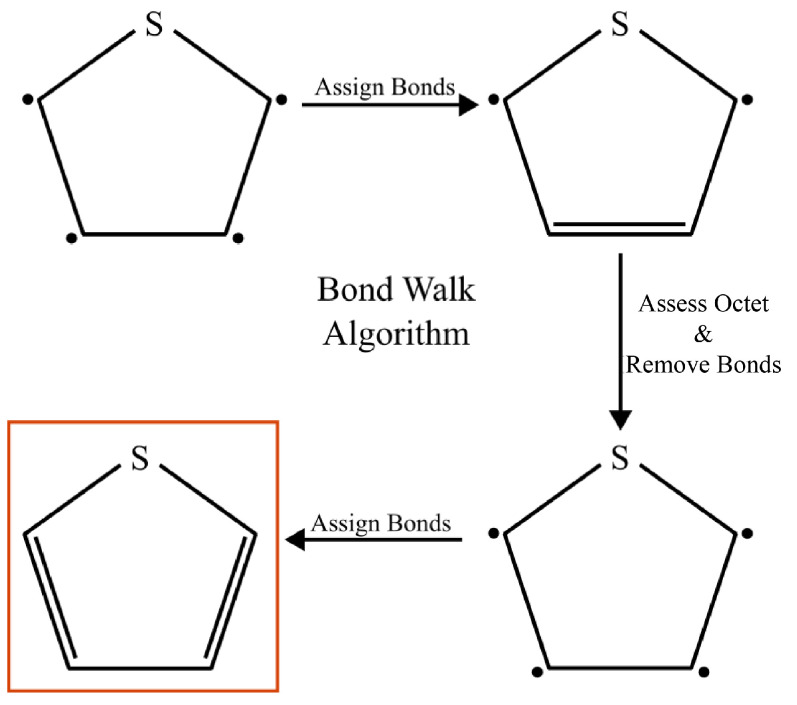
The double- and triple-bond information that is missing from the mBuild compounds is retrieved via BondWalker by iteratively checking whether the octet rules can be satisfied for all atoms after incrementing the bond character adjacent to atoms with unsatisfied octets. Highlighted ring in the lower left shows correct double-bond assignment after application of Bondwalker.

The general procedure was as follows: (1) Create an openMM Molecule topology from the connectivity information of the mBuild Compound to be parameterized. (2) Rebuild the missing double and triple bonds in the openMM Molecule using the BondWalker function (see Figure 13). (3) Pass this “BondWalked” molecule to Espalomafor forcefield parameterization, and then write these parameters to a forcefield xml file. (4) Use the atom labels given by Espaloma to rename the atoms in our mBuild Compound. This last step ensures that the correct parameters will be applied to the corresponding atoms. A full tutorial of Espaloma forcefield and typed mBuild Compound generation can be found at https://github.com/madilynpaul/Espaloma-Validation.

In sum, programmatically generating a molecular representation of perylene or P3HT, with accompanying forcefield definitions that can be used in an MD simulation with the present workflow, requires approximately one minute on a 2014 i7 Macbook pro. In contrast, performing the energy minimizations and constraint sweeps to fill in the missing parameters, as performed in Ref. [22], can take an order of days on a multicore high-performance computing node.

## 5. Conclusions

We successfully incorporated Espaloma forcefield generation into MoSDeF workflows for the purpose of investigating organic molecule phase behavior via MD simulations. Espaloma generates reasonable forcefield parameters for macromolecules with high aromaticity, as well as for thiophene-based conjugated polymers, as measured by GIXS, RDF, and the phase behavior, though energy-scale differences did give rise to quantifiable differences in the local packing and phase transition temperatures. The GIXS scattering patterns for both perylene and P3HT showed a long-range order that is consistent with currently published experimental work, and, in the case of perylene, they have better agreement than OPLS-UAin matching the length scale of the 100 reflection. One caveat is that the absolute energy units generated by ESP-UA may have been too low for the molecules of interest. Here, we found that multiplying ESP-UA ϵ values by 1.26 was in closer agreement with OPLS-UA, which had a better agreement with the P3HT and perylene’s experimental phase transitions between the ordered and disordered phases. We expect other chemistries to have rescaling factors that depend on the ratio between the strongest interaction energies between forcefields being compared, though verifying and quantifying the breadth of other energy-offsets will be reserved for future work. Nevertheless, the present observations inform confidence in using Espaloma to quickly generate forcefields for molecules that are missing information in “off-the-shelf” forcefields like OPLS-UA and GAFF to provide qualitative insight into the packing and phase behavior that are subject to the energy-rescaling caveat made above. We conclude that Espaloma holds promise as a component in the high-throughput screenings of organic molecules for the phase behavior in systems from polymer thermoplastics to organic photovoltaics to macromolecular drug packing and more.

## Figures and Tables

**Table 1:** Lennard–Jones diameters σ, the well-depths ϵ generated by espaloma in the present work, and OPLS-UA and GAFF.

	σ (Å)			ϵ (kJ/mol)		
	**C1**	**C0**	**S**	**C1**	**C0**	**S**
Espaloma	3.481	3.380	3.564	0.3635	0.4554	1.046
OPLS-UA	3.436	3.905	3.436	0.4602	0.7113	1.339
GAFF	3.3997	3.3997	3.564	0.3598	0.4577	1.046

**Table 2:** Summary of the thermodynamic statepoints (defined by T and density) and key simulation parameters for P3HT and perylene.

	P3HT	Perylene
Temperature Range (K)	[60.4, 304.4, 427.7, 608.9]	[49.8, 248.8, 497.7, 746.5]
Density Range (g/cm^3^)	[0.25, 0.5, 1.0]	[0.5, 1.0, 1.5]
N	100	250
dt	0.0003	0.0001
M (amu)	32.06	12.011
σ (Å)	3.56	3.40
ϵ (kJ/mol)	1.046	0.360
N_monomers_	15	1

## Data Availability

Example data and submission scripts for generating all data are available at https://github.com/madilynpaul/Espaloma-Validation.
